# Age-dependent uncoupling of mitochondria from Ca^2+^ release units in skeletal muscle

**DOI:** 10.18632/oncotarget.6139

**Published:** 2015-10-16

**Authors:** Laura Pietrangelo, Alessandra D'Incecco, Alina Ainbinder, Antonio Michelucci, Helmut Kern, Robert T. Dirksen, Simona Boncompagni, Feliciano Protasi

**Affiliations:** ^1^ CeSI - Center for Research on Aging & DNICS, Department of Neuroscience, Imaging and Clinical Sciences, University G. d'Annunzio, Chieti, Italy; ^2^ Department of Pharmacology and Physiology, University of Rochester School of Medicine and Dentistry, Rochester, NY, USA; ^3^ Ludwig Boltzmann Institute of Electrical Stimulation and Physical Rehabilitation & Institute of Physical Medicine and Rehabilitation, Wilhelminenspital, Vienna, Austria

**Keywords:** Ca^2+^ signalling, excitation-contraction coupling, sarcopenia, sarcoplasmic reticulum, triad, Gerotarget

## Abstract

Calcium release units (CRUs) and mitochondria control myoplasmic [Ca^2+^] levels and ATP production in muscle, respectively. We recently reported that these two organelles are structurally connected by *tethers*, which promote proximity and proper Ca^2+^ signaling. Here we show that disposition, ultrastructure, and density of CRUs and mitochondria and their reciprocal association are compromised in muscle from aged mice. Specifically, the density of CRUs and mitochondria is decreased in muscle fibers from aged (>24 months) vs. adult (3-12 months), with an increased percentage of mitochondria being damaged and misplaced from their normal triadic position. A significant reduction in tether (13.8±0.4 vs. 5.5±0.3 tethers/100μm^2^) and CRU-mitochondrial pair density (37.4±0.8 vs. 27.0±0.7 pairs/100μm^2^) was also observed in aged mice. In addition, myoplasmic Ca^2+^ transient (1.68±0.08 vs 1.37±0.03) and mitochondrial Ca^2+^ uptake (9.6±0.050 vs 6.58±0.54) during repetitive high frequency tetanic stimulation were significantly decreased. Finally oxidative stress, assessed from levels of 3-nitrotyrosine (3-NT), Cu/Zn superoxide-dismutase (SOD1) and Mn superoxide dismutase (SOD2) expression, were significantly increased in aged mice. The reduced association between CRUs and mitochondria with aging may contribute to impaired cross-talk between the two organelles, possibly resulting in reduced efficiency in activity-dependent ATP production and, thus, to age-dependent decline of skeletal muscle performance.

## INTRODUCTION

Sarcopenia, age-related decline in skeletal muscle mass and function [1-2], has a major impact on the mobility, quality of life, and health care costs of the elderly [3-4]. As average life expectancy in humans is increasing, a deeper understanding of the mechanisms responsible for impaired muscle function in aging is needed to prevent disabilities, improve independence, enhance quality of life, and reduce health care costs.

Skeletal muscle contraction requires Ca^2+^ and ATP and, thus, depends on the proper function of the following two intracellular organelles: Ca^2+^ release units (CRUs) [5] and mitochondria [6]. CRUs are specialized intracellular junctions, also called *triads,* because they are formed by three elements: a central transverse tubule (TT) flanked by two terminal cisternae (TC) of the sarcoplasmic reticulum (SR). The junctional face of the TC adjacent to the TT membrane bears arrays of ryanodine receptors (RYRs), which function as Ca^2+^ release channels of the SR. CRUs are the site of excitation-contraction (EC) coupling, the process by which membrane depolarization in the TT membrane triggers Ca^2+^ release from the SR [7]. Mitochondria are the powerhouse of the cell, as they are responsible for aerobic production of the majority of cellular ATP used to support various cellular functions in muscle (e.g. cross bridge cycling, active Ca^2+^ transport, etc.). Impaired EC coupling function in aged muscle results in a reduced supply of Ca^2+^ ions to the contractile elements, and thus, reduced specific force [8]. We reported remodeling of triads and a significant decrease in density of CRUs in human biopsies from aged individuals [9-10]. Changes in mitochondrial structure, function, and number have also been suggested to play an important role in age- and disease-related decreases in muscle performance [11-14]. Recent evidence indicates that CRUs and mitochondria are functionally linked to each other via Ca^2+^ and reactive oxygen species (ROS) mediated cross-talk [15-16]. Driven by the large electrochemical gradient across the mitochondrial inner membrane (>-180 mV), a small fraction of Ca^2+^ ions released from the SR during EC coupling is able to enter the mitochondrial matrix through the mitochondrial Ca^2+^ uniporter, or MCU [17-20]. Whereas the physiological relevance of mitochondrial Ca^2+^ uptake in skeletal muscle has been subject to controversy, an increase in Ca^2+^ concentration in the mitochondrial matrix is proposed to stimulate several dehydrogenases and the respiratory chain to increase mitochondrial ATP and ROS production [21-22]. Direct measurements of activity-dependent mitochondrial Ca^2+^ uptake using targeted Ca^2+^ probes have demonstrated that mitochondria sequester Ca^2+^ during cytoplasmic Ca^2+^ oscillations in many different cell types including skeletal muscle fibers [17-18, 23-25].

We previously demonstrated that CRUs and mitochondria in fast twitch skeletal muscle fibers are linked to one another by small strands, or *tethers*, which keeps mitochondria associated to the TC of triads on the side opposite to TTs [26]. In this position, the nearest mitochondrial outer membrane is on average about 160 nm from sites of Ca^2+^ release, i.e. RYR arrays in CRUs located between the TC and TT. During repetitive, high frequency tetanic stimulation, mitochondrial Ca^2+^ uptake mechanisms respond primarily to changes in global Ca^2+^ rather than a highly-privileged and local SR-mitochondrial Ca^2+^ microdomain [17-18]. Association and cross-talk between CRUs and mitochondria may be altered in certain developmental and/or pathological states [26-32]. In addition, we have shown how tethering of mitochondria to CRUs is crucial for the ability of mitochondria to suppress osmotic shock-induced Ca^2+^ spark activity from the adjacent CRU [17], potentially via enhanced local ROS detoxification [33].

As reduced resistance to fatigue is a major concern in the elderly, we quantified tethering of mitochondria to CRUs in mouse *extensor digitorum longus* (EDL) muscles, a fast-twitch muscle in which mitochondria are primarily positioned at the I band in close association to CRUs [26], in adult (3-12 months old) and aged (>24 months old) wild type (WT) mice. Our results reveal a reduction in the density of CRUs, mitochondria and tethers, reduced activity-dependent mitochondrial Ca^2+^ uptake, and increased oxidative stress in skeletal muscle from aged mice.

## RESULTS

We compared two populations of wild type (WT) male C57bl/6 mice: 3-12 months old (referred to as adult) and >24 months old (referred to as aged). While average body weight was significantly increased in aged mice (Fig. S1, left), peak grip strength was significantly reduced (Fig. S1, right). This decrease in grip strength indicates a general impairment in neuromuscular function in mice of advanced age.

### a Light, confocal, and electron microscopy examination of EDL muscle fibers (Figures 1, 2 and S2)

**Figure 1:**
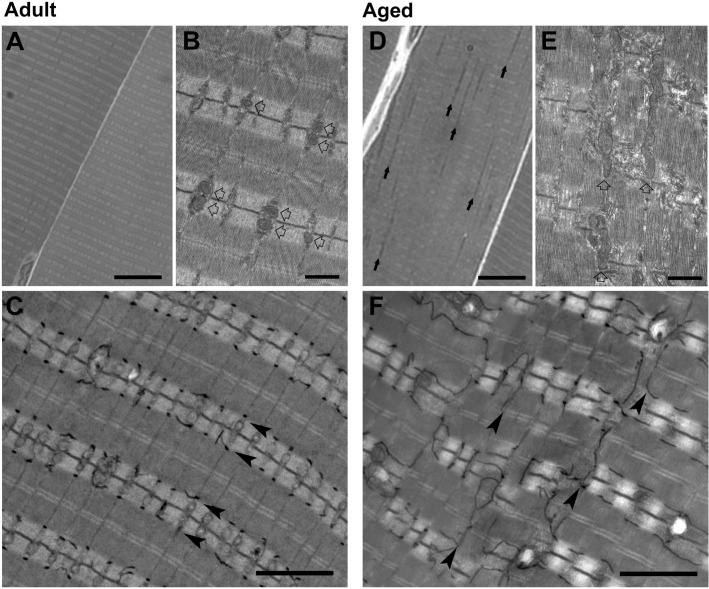
Analyses of adult and aged EDL muscle by light and electron microscopy (EM) **A.**-**C**. Adult fibers are characterized by an extremely ordered transversal pale-dark striation (A; histological section), with most mitochondria positioned within the I-band of the sarcomere, as best visualized at higher EM magnification (empty arrows in B). TTs (stained in black in C) are almost exclusively located at the level of the A-I junction (arrowheads in C). **D.**-**F.** In fibers from aged mice, striations are not as well preserved as in adult fibers (D; histological section), with some mitochondria forming longitudinal columns between myofibrils (arrows in D and arrowheads in E). Sarcomeres are often not correctly aligned with one another (E). Misalignment of contractile elements (E) also impacts TTs (stained in black in F), which are often longitudinally oriented (arrowheads in F). Scale bars: A and D, 10 μm; B and E, 1 μm; C and F, 2 μm.

Light and electron microscopy (EM) analyses of semi-thin and thin sections at low-medium magnification revealed that the overall ultrastructure of muscle fibers was less ordered in EDL muscle from aged mice compared to that of adult mice (Fig. 1). Specifically, fibers from adult mice were characterized by readily visible and regular transverse pale-dark striations (Fig. 1A). Most mitochondria were positioned within the I-band of the sarcomere, as best visualized at higher EM magnification (Fig. 1B, empty arrows). In fibers from aged mice, on the other hand, striations were not as sharp (Fig. 1D) and mitochondria were more often found in longitudinal columns between myofibrils (Fig. 1D, arrows; Fig. 1E, empty arrows). Also, myofibrils in muscle fibers from aged mice were often not well-aligned laterally with Z lines of adjacent sarcomeres (Fig. 1E). This loss of organization in aged fibers also impacted the structure of the sarcotubular system (stained in black in Fig. 1C and 1F): indeed, TTs, almost exclusively located at the A-I junction in adult muscle (Fig. 1C, arrowheads), exhibited areas of partial disarray and longitudinal orientation in muscle fibers from aged mice (Fig. 1F, arrowheads).

**Figure 2:**
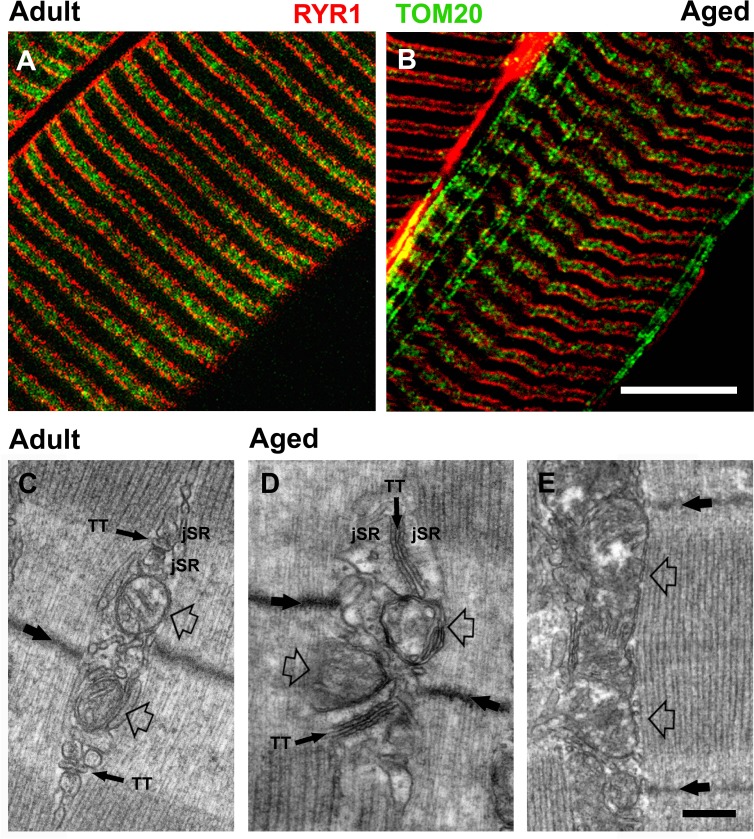
Confocal and electron microscopy (CM and EM) of CRUs and mitochondria **A.** and **B**. Double immunolabeling of RyR1 (in red) and of TOM20 (in green) marks the position of CRUs and mitochondria, respectively. In fibers from aged mice, cross striations are partially lost with some mitochondria staining exhibiting a longitudinal orientation. **C**. In muscle fibers from adult mice, CRUs are located on both sides of the Z line (large back arrow), contain a TT element that is oriented transversally with respect to the main axis of the surrounding myofibrils, and are also often in close proximity to a mitochondrion (empty arrows). **D**. and **E**. In fibers from aged mice, the disposition, morphology, and orientation of both CRUs and of mitochondria are often compromised, with triads exhibiting an oblique and/or longitudinal orientation (D) and some mitochondria extending longitudinally between myofibrils (E, empty arrows). Scale bars: A and B, 5μm; C-E, 0.25μm.

We immunolabeled proteins of the CRU (type I RYR or RYR1) and mitochondria (20 kD subunit of the translocase outer membrane or TOM20) to visualize structural modifications of triads and mitochondria by confocal microscopy (CM) (Fig. 2 and S2). CM confirmed a disrupted disposition of CRUs and mitochondria in muscle fibers from aged mice (Fig. 2A and 2B). In adult mice, CRU and mitochondrial double-immunolabeling revealed typical double-row striations of CRUs with adjacent mitochondria located within the I band region of the sarcomere (Fig. 2A and Fig. S2A and S2B). On the other hand, this precise registration of CRUs and mitochondria was partially lost in fibers from aged mice (Fig. 2B and Fig. S2C and S2D). In some regions of fibers from aged mice, mitochondria also formed clusters and/or longitudinal rows between myofibrils (Figs. 2B and S2F), likely in correspondence of areas exhibiting disrupted or misplaced CRUs (arrow in Fig. S2E and enlarged inset).

Analyses of EDL fibers by EM at higher magnification (Fig. 2C-2E) confirmed that CRUs in adult fibers: a) were located on both sides of the Z line (Fig. 2C, thick back arrow); b) exhibited TTs oriented transversally with respect to the main axis of the surrounding myofibrils; and c) were typically in close proximity to a single mitochondrion (Fig. 2C, empty arrows) located on the I-band side of the CRU (see also [26] for more detail). In fibers from aged mice, however, the disposition and orientation of both CRUs and mitochondria were often compromised, exhibiting an oblique or even longitudinal orientation (Fig. 2D) and mitochondria often extended longitudinally between myofibrils (Fig. 2E, empty arrows). Also, mitochondrial morphology was often abnormal, appearing swollen and/or structurally damaged (Fig. 2D and 2E).

### b Quantitative analyses of CRUs, mitochondria, and tethers (Tables 1, 2, and S1; Figures 3 and S3)

We quantified the density and positioning of CRUs and mitochondria with respect to sarcomere orientation in electron-micrographs of muscle fibers from adult and aged mice (Tables 1 and 2). These analyses revealed that the density of CRUs and mitochondria were significantly reduced (about 15-20%) in aged compared to that observed in adult mice (columns A in Tables 1 and 2). We also found that the relative fiber volume occupied by mitochondria was significantly reduced (~10%) in fibers from aged mice (Table 2, column B). Moreover, the position and orientation of CRUs and mitochondria were altered in fibers from aged mice, as indicated by a significant increase in: a) oblique and longitudinal CRUs (Table 1, columns B and C; see also Fig. S3); b) number of dyads or incomplete CRUs (Table 1, column D); and c) aberrant localization of mitochondria at the A band (Table 2, column C; see also Fig. 2E). We also quantified the percentage of mitochondria exhibiting structural abnormalities such as translucent matrix, cristae remodeling and/or disruption, vacuoles, and multi-layered myelin figures. Severely altered mitochondria were more frequently observed in aged fibers (Table 2, column D).

**Table 1:** Quantitative analyses of density and orientation of CRUs

	A	B	C	D
	No. of CRUs/100 μm^2^	% of Oblique CRUs	% of Longitudinal CRUs	% of Dyads
Adult	87.4 ± 1.3	3.3 ± 0.4	0.2 ± 0.04	0.5 ± 0.1
Aged	74.1 ± 1.1*	8.7 ± 0.8*	0.9 ± 0.2*	4.0 ± 0.4*

Data in column A are shown as mean ± SEM (*p < 0.01).

Samples size: 50 fibers from 5 adult mice and 50 fibers from 5 aged mice; 10 micrographs/fiber

**Table 2:** Quantitative analyses of mitochondrial density and intracellular disposition

	A	B	C	D
	No. of Mitochondria /100 μm^2^	Mitochondrial Volume/Total Volume (%)	No. of Mitochondria at A band / 100 μm^2^ (%)	Severely Altered Mitochondria (% of total)
Adult	52.9 ± 1.1	8.8 ± 0.5	1.5 ± 0.1 (3%)	4.7 ± 0.1
Aged	42.8 ± 0.9*	8.0 ± 0.6*	5.9 ± 0.2 (14%)*	14.9 ± 0.1

Data in column A are shown as mean ± SEM. ( *p < 0.01).

Samples size: 50 fibers from 5 adult mice and 50 fibers from 5 aged mice; Columns A and C, 10 micrographs/fiber; Column B: 2 micrographs/fiber (low mag images in cross section).

**Figure 3:**
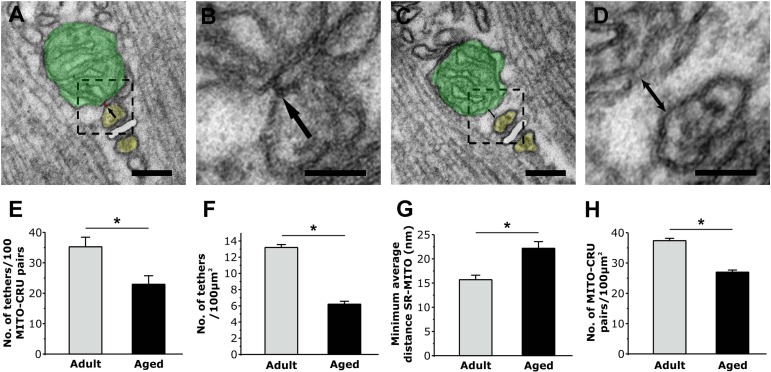
Quantitative analyses of tether density and CRU-mitochondrial association **A.** and **B**. Tethers are small electron dense strands linking the outer mitochondrial membrane to a TC of the triad (arrows). **C**. and **D**. When tethers are absent, the distance between mitochondria and SR increases (double headed arrow). **E**. and **F**. The number of tethers observed in 100 mitochondria-CRU pairs (E) and the calculated number of tethers / 100μm^2^ (F) significantly decreases in fibers from aged mice. **G**. The average minimum mitochondrion-SR distances increased in fibers from aged mice. **H**. The number of CRU-mitochondrion pairs / 100μm^2^ is significantly decreased in fibers from aged mice. Colours legend: TC of the SR in yellow; TT in white; mitochondria in green. Data are plotted as mean ± SEM (*p<0.01). Scale Bars: A and C, 0.1μm; B and D, 0.05 μm.

The association of mitochondria to CRUs is mediated by *tethers,* small electron-dense strands that link the two intracellular organelles together (Fig. 3A and 3B, arrows), the incidence of which is markedly increased during post-natal maturation [26]. We quantified tether density and the minimum average distance between SR and mitochondrial membranes in EDL fibers from adult and aged mice (Fig. 3 and Table S1): both the number of tethers in 100 mitochondria-CRU pairs and the number of tethers/100μm^2^ were significantly decreased in fibers from aged mice (Fig. 3E and 3F; see Table S1 for more detail). In addition to the reduction in tether number, the minimum average distance between adjacent mitochondrial and SR membranes (Fig. 3C and 3D, double-headed arrow) was significantly increased in fibers from aged mice compared to that of fibers from adult mice (Fig. 3G). As the coupling of mitochondria to the CRU depends on the relative density of the two elements (Tables 1 and 2) and tethers (Fig. 3E and 3F), we also quantified the number of CRU-mitochondria pairs per 100μm^2^ (Fig. 3H). As expected, the density of mitochondrion-CRU pairs was significantly reduced in fibers from aged mice (~30%).

### c Measurements of 3-nitrotyrosine (3-NT) levels, SOD1 and SOD2 expression (Figure 4)

**Figure 4:**
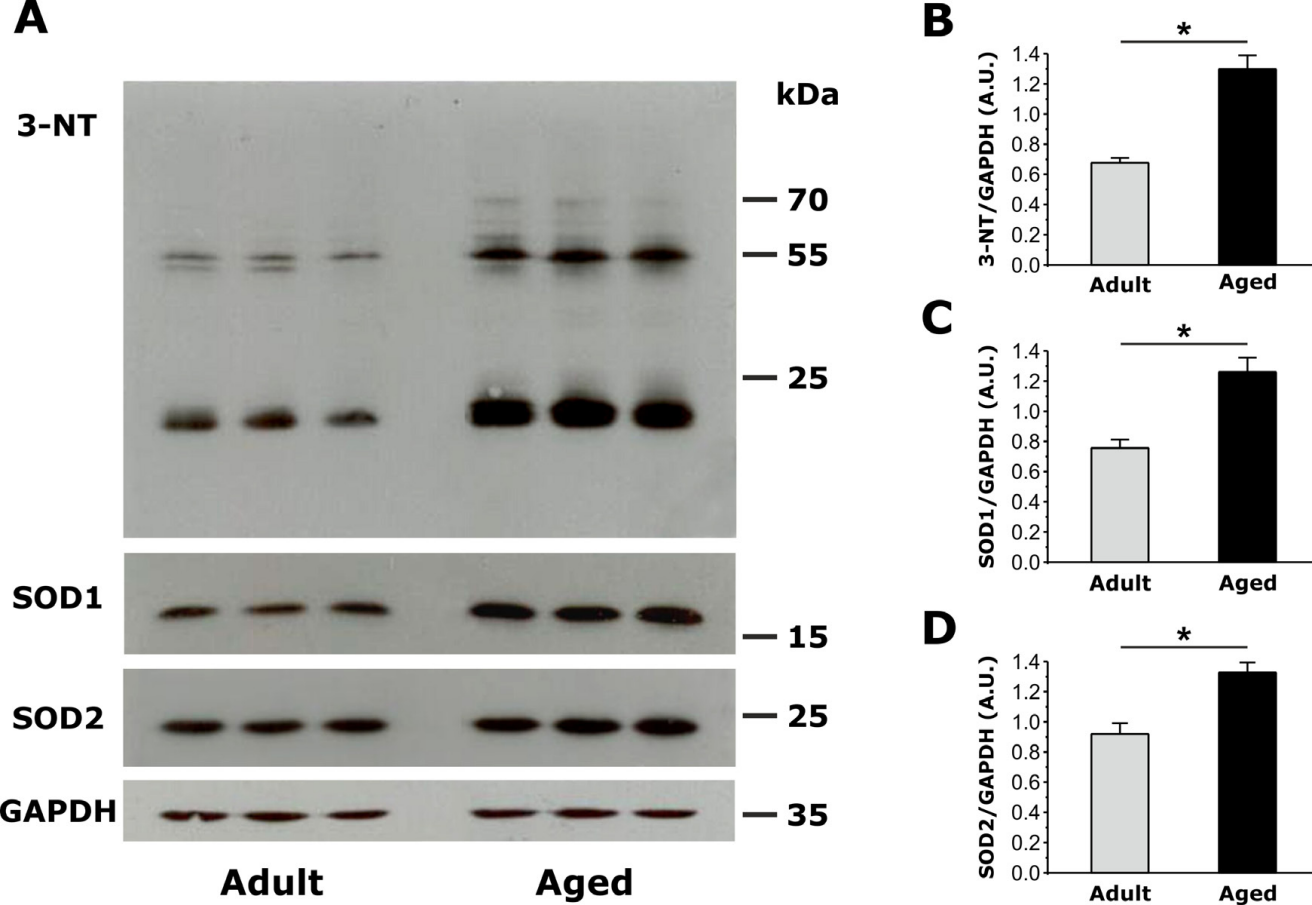
Assessment of oxidative stress, measured as the amount of total 3-Nitrotyrosine (3-NT), copper/zinc superoxide dismutase (SOD1) and manganese superoxide dismutase (SOD2) expression in EDL muscles from adult and aged mice **A**. Representative immunoblots showing expression levels of 3-NT, SOD1 and SOD2 in EDL muscles from adult (n=4, only 3 shown) and aged (n=4, only 3 shown) mice. **B**.-**C**. Relative abundance of 3-NT, SOD1 and SOD2 normalized on GAPDH band densities, reveals that both markers of oxidative stress are significantly increased in EDL muscles of aged mice compared to that observed in adult mice. Data are given as means ± SEM (*p<0.01).

Oxidative stress is elevated in aging muscle [34]. To compare oxidative stress in EDL muscles from adult and aged mice, we measured: a) levels of 3-nitrotyrosine (3-NT), the nitration of protein tyrosine residues mediated by reactive species of nitrogen (RNS) such as peroxynitrite anion and nitrogen dioxide; b) expression of Cu/Zn superoxide dismutase (SOD1, the myoplasmic isoform) and Mn superoxide dismutase (SOD2, the mitochondrial isoform), which catalyze the transformation of superoxide (O_2_^−^) into oxygen (O_2_) and hydrogen peroxide (H_2_O_2_), the first step for elimination of reactive oxygen species (ROS). Western blot analyses revealed that 3-NT levels, a biomarker of oxidative stress and mitochondrial damage, were significantly increased in EDL muscles from aged mice (Fig. 4A and 4B). In the same samples, SOD1 and SOD2 expression (relative to GAPDH) were also significantly increased in EDL muscles from aged mice (Fig. 4A and 4C).

### d Measurement of activity-dependent mitochondrial Ca^2+^ uptake in FDB fibers from adult and aged mice (Figures 5, 6 and S4)

**Figure 5:**
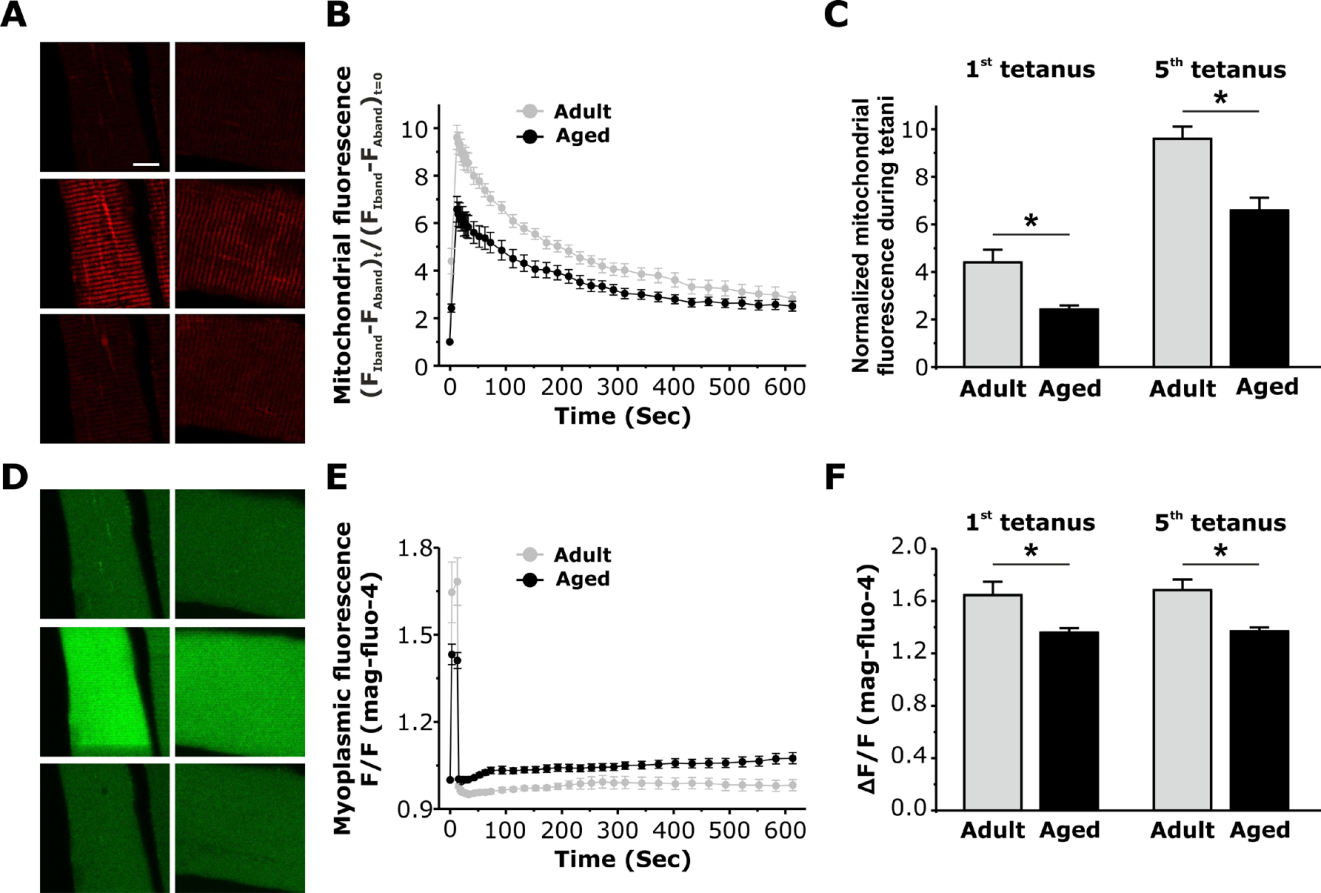
Measurement of activity-dependent mitochondrial Ca^2+^ uptake and myoplasmic Ca^2+^levels in FDB fibers from adult and aged mice **A**. Representative images of rhod-2 fluorescence in FDB fibers from adult and aged mice before, during tetanic stimulation (5^th^ tetani), and 9 minutes after the 5^th^ tetanus. **B**. Average time course of rhod-2 fluorescence in FDB fibers at rest, during 5 successive tetanic stimuli, and for 9 minutes of recovery following the 5^th^ and final tetanus in FDB fibers from adult (black circles) and aged (grey circles) mice. **C**. Average peak normalized rhod-2 fluorescence in FDB fibers during the 1^st^ and the 5^th^ tetanic stimuli in FDB fibers from adult and aged mice shows a significant reduction in peak mitochondrial Ca^2+^ uptake in fibers from aged mice. **D**. Representative mag-fluo-4 fluorescence images in FDB fibers from adult and aged mice during a 500ms tetanus. **E**. Average relative mag-fluo-4 fluorescence in FDB fibers from adult and aged mice at rest, during tetanic stimulation (5^th^ tetani), and for 9 minutes of recovery following the 5^th^ and final tetanus in FDB fibers from adult (black circles) and aged (grey circles) mice. **F**. Average mag-fluo-4 fluorescence in FDB fibers during 1^st^ and the 5^th^ tetanic stimuli in FDB fibers from adult and aged mice shows a significant reduction in the peak myoplasmic Ca^2+^ transient in fibers from aged mice. Data are plotted as mean ± SEM (* p<0.01).

Since the density, positioning, and tethering of mitochondria and CRUs were altered in fibers from aged mice, we measured the ability of mitochondria to sequester Ca^2+^ following a train of five consecutive high frequency tetanic stimulations (500 ms, 100 Hz, 0.2 duty cycle) [17]. Mitochondrial Ca^2+^ uptake after both the first and fifth tetanus was significantly reduced in fibers from aged mice compared to that observed in fibers from adult mice (Fig. 5A-5C). Consistent with previous studies [8], a significant reduction in the magnitude of the peak global myoplasmic Ca^2+^ transient during both the first and fifth tetanus was also observed in fibers from aged mice (Fig. 5D-5F).

**Figure 6:**
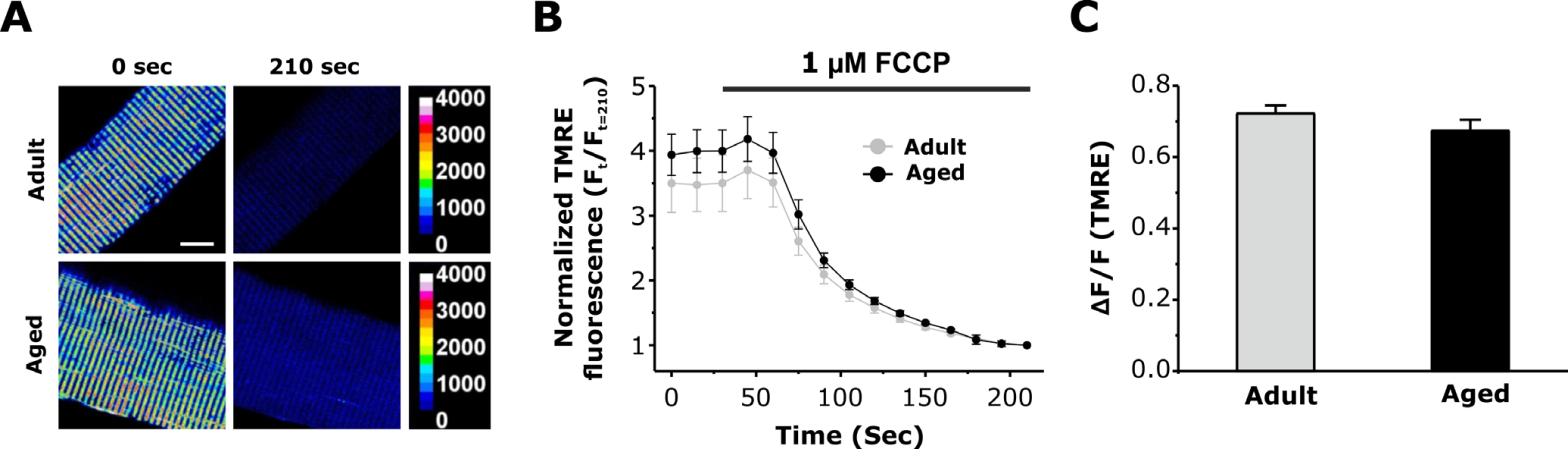
Measurement of mitochondrial membrane potential in FDB fibers from adult and aged mice **A**. Representative pseudocolor TMRE confocal images of FDB fibers from adult and aged mice before and after application of 1μM FCCP. Scale bars on the right represent relative pseudocolor fluorescence intensities. **B**. Average normalized TMRE fluorescence of FDB fibers from adult and aged mice before and during application of 1 μM FCCP (grey bar). **C**. Average maximal FCCP-induced change in TMRE fluorescence was not significantly different in FDB fibers from adult and aged mice. Data are plotted as mean ± SEM (*p<0.01).

The mitochondrial membrane potential (ΔΨ_m_) is a critical determinant of the driving force for mitochondrial Ca^2+^ uptake. To determine if a reduction in ΔΨ_m_ contributes to the observed decrease in activity-dependent mitochondrial Ca^2+^ observed in fibers from aged mice, we quantified the FCCP-sensitive change in ΔΨ_m_ in FDB fibers loaded with 20nM TMRE. FDB fibers exhibited clear double rows of transverse TMRE fluorescence, consistent with efficient loading of the dye into mitochondria located within the I band (Fig. 6A, left panels). Subsequent addition of 1 μM FCCP resulted in a time-dependent dissipation of TMRE fluorescence (Fig. 6A, right panels and Fig. 6B). The magnitude of FCCP-dependent dissipation of ΔΨ_m_ was not different between fibers from adult and aged mice (Fig. 6C). Western blot analyses revealed that the levels (relative to GAPDH) of some mitochondrial and Ca^2+^ handling proteins (MCU, Mfn2, and VDAC, and RYR1) were not significantly different in FDB muscles from adult and aged mice (Fig. S4).

## DISCUSSION

### Main findings

**Figure 7:**
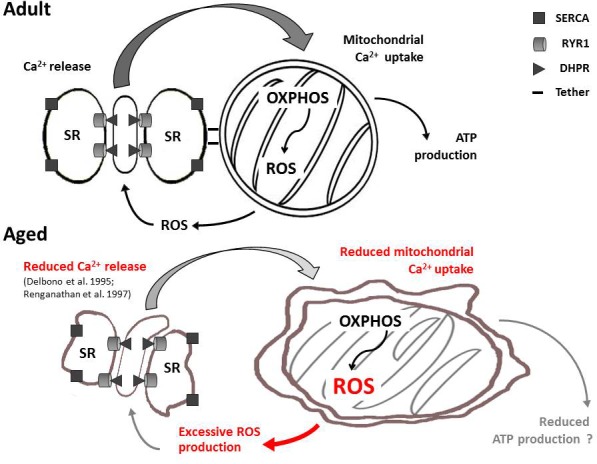
Model summarizing age-related changes to CRU-mitochondrial cross-talk Aging causes changes in the morphology and relative positioning of CRUs and mitochondria. These structural changes are accompanied by functional alterations including reduced Ca^2+^ release from CRUs (see Fig. 5D-5F and [8, 41]), reduced mitochondrial Ca^2+^ uptake (Fig. 5A-5C) and excessive production of reactive oxidative species (Fig. 4). It remains to be determined if changes in mitochondrial distance from CRUs and increased ROS production influences Ca^2+^ release from the triad (e.g. via altered Ca^2+^ buffering and redox regulation of RYR1) and activity-dependent aerobic ATP production, and hence, muscle performance.

Between 2008 and 2011, we published a series of papers investigating the functional and structural cross-talk between CRUs and mitochondria in skeletal muscle fibers [15, 17, 35-36]. These studies revealed how these two organelles are anchored together by tethers [26] and that this association is altered in several pathophysiological states [27-30, 32]. Here we evaluated the association and functional cross-talk between CRUs and mitochondria in muscle fibers from aged mice. Our results reveal that the density of CRUs, mitochondria, and tethers decrease with age (Tables 1, 2, and S1; Fig. 3), resulting in loss of proper organization, with an increased percentage of both organelles becoming longitudinally oriented between myofibrils (Figs. 1, 2, S2, and S3; Tables 1 and 2). The changes in density, orientation, and disposition of CRUs and mitochondria, as well as the disassociation of some mitochondria from CRUs with age are modelled in Fig. S5. These structural changes likely contribute to impaired function including reduced myoplasmic Ca^2+^ transients, decreased mitochondrial Ca^2+^ uptake, and excessive production of reactive oxygen species (Figs. 4 and 5). In Fig. 7, we propose a model that integrates the age-dependent structural and functional changes observed in this study with prior published results (i.e. reduced EC coupling [8, 37-41]) that could lead to inefficient activity-dependent mitochondrial ATP production and, hence, reduced muscle performance at advanced ages. The different aspects of this model are discussed in detail in the following paragraphs.

### Loss of proper position and reciprocal association of CRU and mitochondria

Dysfunctional EC coupling [8, 41], reduced density of CRUs [9], and changes in mitochondrial structure and function have been suggested to play an important role in age-related reductions in muscle performance [12-13]. Data presented here not only confirm age-related changes in CRU and mitochondrial density and structure (Figs. 1, 2, S2, and S3 and Tables 1 and 2), but also focus the attention on alterations in position, orientation, and reciprocal association of these two intracellular organelles. The observed changes in CRU and mitochondrial density and disposition (Tables 1 and 2) may account in part for the observed significant reduction (about 30%) in the density of CRU-mitochondria pairs in aged fibers (Fig. 3 and Table S1). A similar reduction in density of CRU-mitochondria pairs was also reported in biopsies obtained from elderly humans [10]. Indeed, together with previous findings [9-10], these results suggest that age-dependent changes in the association between membrane systems and organelles involved in EC coupling (CRUs) and aerobic metabolism (mitochondria) may interfere with proper CRU-mitochondrion cross-talk (Fig. 7) required for efficient activity-dependent aerobic ATP production (required for optimal force generation and muscle performance) and local ROS signaling [15, 17, 33].

However, the molecular mechanisms that underlie the physical uncoupling of CRU-mitochondrion pairs in aged muscle remains to be determined. We previously proposed that tethers anchor mitochondria to the triad [28]. Indeed, we found that CRU-mitochondrial tether density is decreased (Fig. 3E and 3F) and the average minimum distance between these two organelles is increased in fibers from aged mice (Fig. 3G). However, whether a reduction in tether density is responsible for the detachment and movement of mitochondria away from CRUs, or if loss of tethers is the result of uncoupling, remains unclear. While we recently proposed that mitofusin-2 (Mfn2) may be involved in tethering mitochondria to the CRU [18], Mfn-2 levels were not altered in muscle from aged mice (Fig. S4).

### Reduced myoplasmic Ca^2+^ elevation and mitochondrial Ca^2+^ uptake

We found that activity-dependent increases in myoplasmic mag-fluo-4 fluorescence and mitochondrial rhod-2 fluorescence were both similarly reduced (~20-30%) in FDB fibers from aged mice (Fig. 5). Our finding of reduced myoplasmic Ca^2+^ levels following stimulation confirms the extensive work of Delbono and colleagues clearly showing impaired EC coupling in fibers from aged mice [8, 37-41]. We also previously suggested that this reduction in stimulation-induced Ca^2+^ release may also depend on a significant age-dependent loss of CRUs [9], a finding confirmed in the present study (Table 1). Rudolf et al (2004) reported fast mitochondrial Ca^2+^ transients in response to both twitch and tetanic stimulation in fast twitch fibers from EDL muscle [42]. We subsequently confirmed transient elevations in mitochondrial Ca^2+^ during twitch stimulation in isolated FDB fibers expressing mitochondrial-targeted pericam [17]. Thus, the observed reduction in mitochondrial Ca^2+^ uptake in fibers from aged mice (Fig. 5) is most likely due to the reduced global myoplasmic Ca^2+^ transients generated in these fibers (Fig. 5 and [37-41]). Neverheless, our findings also suggest that a reduced density of mitochondria (Table 2) and an increased separation between CRU and mitochondria (Fig. 3 and Table S1) could also contribute to the age-dependent reduction in mitochondrial Ca^2+^ uptake.

### Increased oxidative stress, a possible factor causing damage to cellular organelles

Nitration of protein tyrosine residues (3-NT), as well as SOD1 and SOD2 ([43] expression were both increased in skeletal muscle from aged mice (Fig. 4), consistent with increased oxidative stress in sarcopenia as reported previously [12, 34]. Subsequent oxidative modifications to critical cellular proteins, membrane lipids, cytoskeleton, etc. [12, 34] could result in structural modifications to CRUs and mitochondria (Figs. 1, 2, 3, S2 and S3) that, in turn, would lead to improper Ca^2+^ handling, mitochondrial respiration, and ROS production (Fig. 7). Ultimately, these events could drive a dangerous feed-forward mechanism that further exacerbates oxidative cellular damage that eventually will involve all other critical components, including the contractile machinery. The augmented expression of SOD1 likely reflects a compensatory response to excessive production of ROS, a finding in agreement with the observation that SOD1 expression and activity are increased under conditions of high oxidative stress both in aging and under physio-pathological conditions [44-45]. On the contrary, as conflicting results on SOD2 functionality in aging have been reported [46], the increased expression of SOD2 observed in our experiments and the precise role of SOD2 in aging requires further investigation.

### Significance and future directions

This study revealed that the association between CRUs and mitochondria is altered with aging (Fig. S5). It remains to be determined the degree to which changes in mitochondrial distance from CRUs and increased ROS production influence: a) Ca^2+^ release from the triad (e.g. via altered Ca^2+^ buffering and redox regulation of RYR1); and b) efficient ATP production required for activity-dependent force production. However, as proper cross-talk between CRUs and mitochondria may be important for aerobic ATP production in muscle (Fig. 7), our finding of increased mitochondrion-CRU separation suggests that interventions designed to prevent uncoupling of mitochondria from CRUs may improve muscle performance in the elderly, which would significantly enhance mobility, independence, and quality of life. In the future new approaches need to be developed (e.g. exercise, training) that maintain/improve association of mitochondria with CRUs in aged muscle. Indeed studies of biopsies from athletically-active aged individuals [10] indicate that aerobic exercise could be an effective strategy to counteract age-dependent uncoupling of mitochondria from CRUs. However, more rigorous and controlled studies of the impact of exercise intensity and duration on CRU-mitochondrial coupling and muscle function in animal models are needed. In addition, as oxidative stress may underlie damage to subcellular organelles, it will also be important to assess the efficacy of pharmacological and/or dietary interventions to reduce oxidative stress and prevent age-dependent destruction of CRUs/mitochondria cross talk in skeletal muscle fibers.

## MATERIALS AND METHODS

In this study, we compared two populations of male wild type (WT) C57bl/6 mice: 3-12 months old (referred to as adult) and >24 months old (referred to as aged) animals. All experiments were conducted according to the Directive of the European Union 2010/63/UE and the National Institutes of Health *Guide for the Care and Use of Laboratory Animals*, and were approved by the animal ethical committees of all contributing institutions. Mice were housed in micro-isolator cages at 20°C, in a 12hr light/dark cycle, and provided free access to water and food. Mice were sacrificed by cervical dislocation as approved by the local University Committee on Animal Resources (15/2011/CEISA/COM; UCAR-2006-114R). For the purpose of this study, C57bl/6 male mice were divided in two groups of ages: a) adult mice (3-12 months) and b) aged mice (> 24 months).

### Grip strength test

As a standard procedure, weight and measured grip strength were recorded for C57bl/6 mice in different age groups. Force developed by mice during instinctive grasp (i.e. grip strength) was measured as described in [47]. Briefly, mice were held by the tail and lowered to a metal grating connected to the shaft of a Shimpo Fgv 0.5x force transducer (Nidec-Shimpo America Corporation, Itasca, USA). Once the mouse had firmly grabbed the grating, a steady, gentle pull was exerted on the tail. Measurements of peak force generated by each mouse using fore- and hind-limbs were repeated three times with appropriate intervals (at least 30 sec.) to avoid fatigue and the highest value of peak force (normalized to total body mass) measured before each experiment was used.

### Preparation and analysis of samples for light and electron microscopy (EM)

EDL muscles were dissected from sacrificed animals, pinned on a Sylgard dish, fixed at room temperature with 3.5% glutaraldehyde in 0.1 M NaCaCO buffer (pH 7.2), and stored in the fixative at 4°C. Small bundles of fixed muscle were then post-fixed, embedded, stained en-block, and sectioned for EM, as described previously [26, 28]. For TT staining, specimens were post-fixed in a mixture of 2% OsO4 and 0.8% K_3_Fe(CN)_6_ for 1-2 h followed by a rinse with 0.1M sodium cacodylate buffer with 75mM CaCl_2_ [48].

For histological examination, 700 nm thick sections were stained in a solution containing 1% Toludine blue O and 1% Sodium Tetraborate in distilled water for 3 minutes on a hot plate at 55-60°C. After washing and drying, sections were mounted with mounting medium (DPX Mountant for Histology, SIGMA) and observed with a Leica DMLB light microscope (Leica Microsystem, Germany). For EM, ultrathin sections (~50 nm) were examined after staining in 4% uranyl acetate and lead citrate, with a FP 505 Morgagni Series 268D electron microscope (FEI Company, Brno, Czech Republic), equipped with Megaview III digital camera (Munster, Germany) at 60 kV or 100kV (TT staining preparations).

### Quantitative analyses of CRUs and mitochondria

Data contained in Tables 1, 2, and S1 were collected from adult and aged EDL muscles (5 mice for each group). Micrographs of non-overlapping regions were randomly collected from transversal and longitudinal sections of internal fiber areas (see Tables for additional detail about sample size). For all quantifications, fibers containing tubular aggregates were intentionally excluded from the analysis [49].

A) Density of CRUs, mitochondria and mitochondria-CRUs pairs (respectively: Table 1, columns A; Table 2, Column A; Fig. 3H and Table S1, column D) was evaluated in longitudinal sections and reported as average number over 100 μm^2^ (see [26] for more detail). In each EM image, we also determined the orientation of CRUs and percentage of dyads (Table 1, columns B and D, respectively-D) and the mitochondrial positioning with respect to the I and A bands (Table 2, column C). If an individual mitochondrion extended from one I band to another, it was counted in both.

B) Mitochondrial volume (Table 2, column B) was determined using the well-established stereology point-counting technique [50] in micrographs taken from transversal sections at magnification 7.100X. Briefly, after superimposing an orthogonal array of dots at a spacing of 0.20 μm to the electron micrographs, the ratio between numbers of dots falling within mitochondrial profiles and total number of dots covering the whole image was used to calculate the relative fiber volume occupied by mitochondria.

C) The density of tethers connecting SR to the mitochondrial outer membrane and the minimum average distance between SR and mitochondrial membranes were determined as in [26, 28]. The number of severely altered mitochondria was counted and their number reported as percentage of the total number (n=3316 for adult and n=4482 for aged). Mitochondria with any or several of the following ultrastructural alterations were classified as severely altered: b) mitochondria with clear disruption of the external membrane; b) severe vacuolization and disruption of the mitochondria internal cristae; c) mitochondria containing myelin figures (see [28] for more detail).

### Immunolabeling and confocal microscopy (CM)

EDL muscles were dissected from sacrificed mice, fixed in 2% paraformaldehyde in phosphate buffered saline (PBS) for 20 min at RT and stored at 4°C overnight. Smalls bundles of fixed fibers were: a) washed twice with PBS; blocked for 1 hour in PBS containing 1% bovine serum albumin (BSA), 10% goat serum and 0.5% TRITON X-100; b) incubated overnight at 4°C in primary antibody diluted in PBS/BSA; washed three times in PBS; and c) incubated with the secondary antibody for one hour at RT before being mounted on coveslips with anti-bleach media. Primary antibodies used were the following: mouse anti-RYR 34C (1:30; Developmental Studies Hybridoma Bank, University of Iowa); rabbit polyclonal anti-TOM20 (1:100; Santa Cruz). Secondary antibodies used were the following: Cy3-labeled goat anti-mouse IgG diluted 1:300; Cy3-labeled goat anti-rabbit IgG diluted 1:300; Cy5-labeled goat anti-mouse IgG diluted 1:200. All secondary antibodies were from Jackson ImmunoResearch Laboratories, Lexington, KY. Specimens were viewed and imaged using a scanning laser confocal microscope (LSM 510 META Carl Zeiss, Germany) interfaced with an inverted Zeiss Axiovert microscope.

### Western blot analyses

For assessment of 3-nitrotyrosine (3-NT), Cu/Zn superoxide dismutase (SOD1) and Mn superoxide dismutase (SOD2) expression levels, Western blot experiments were performed as previously described [51]. Briefly, EDL muscles obtained from adult (n=4) and aged (n=4) mice were homogenized in SDS 3% and EGTA 1 mM lysis buffer and centrifuged for 15 min at 900 × g at RT. Obtained supernatant was used for supernatant for total protein quantification using a modified Lowry method. 40 μg of total protein were separated in 12% sodium dodecyl sulphate-polyacrylamide gel electrophoresis (SDS-PAGE) gels and transferred to nitrocellulose membrane. Membranes were probed using primary antibodies against SOD1 (1:1000, Santa Cruz Biotecnology) and SOD2 (1:2000, Santa Cruz Biotecnology), 3-Nitrotyrosines (Millipore), and glyceraldehyde-3-phosphate dehydrogenase (GAPDH, 1:10000, OriGene), diluted in 5% non-fat dry milk in TBS-T overnight at 4°C. Horseradish peroxidise (HRP)-conjugated anti-mouse or -rabbit (1:10000, Calbiochem) were used as secondary antibody. Peroxidase activity was detected using an Enhanced chemiluminescence (ECL) kit (Perkin Elmer), and band intensities were analyzed and quantified using Image J software.

For assessment of Ca^2+^ handling and mitochondrial proteins levels, whole FDB and EDL muscles were carefully dissected and homogenized using a Potter homogenizer while on ice in Ripa buffer (1% NP-40, 0.25% NaDOc, 50mM Tris, 150mM HCl, pH 7.4) supplemented with complete protease inhibitors cocktail (Roche, Branford, CT). The homogenate was then solubilized for 1 hr at 4°C with gentle agitation and centrifuged at 13,000 × g for 15 min at 4°C. Total protein concentration in the supernatant was determined using the reducing agent and detergent compatible protein assay (BioRad, Hercules, CA). Protein samples (5-10μg), solubilized in 4X sample buffer (40% glycerol, 0.24M Tris/HCl pH6.8, 0.28M SDS, 0.6mM bromophenol blue, 5% β-mercaptoethanol) were loaded in each lane and separated using either 4%, 10% or 12% polyacrylamide gels in Tris–Glycine running buffer. Proteins were then transferred onto nitrocellulose membranes (BioRad BioRad, Hercules, CA) overnight at room temperature in a buffer containing 25mM Tris Base, 191mM Glycine and 20% methanol. The membranes were then blocked with TBST (20mM Tris, 137mM NaCl 0.75% Tween20) plus 5% Blotting Grade Blocker for 1 hr at room temperature and immunostained with primary antibodies: α-Mfn2 (1:1000, Sigma, St. Louis, MO), α-MCU (1:500, Sigma, St. Louis, MO), α-glyceraldehyde 3-phosphate dehydrogenase (GAPDH, 1:10000, Ambion, Austin, TX), α-dihydropyridine receptor (1:300, Thermo Fisher Scientific, Waltham, MA), α-SERCA1 (1:5000, Santa Cruz, Santa Cruz, CA), α-RYR (34C, University of Iowa, Iowa City, IA), or α-VDAC (1:1000, Millipore, Billerica, MA), followed by incubation with appropriate goat anti-mouse or anti-rabbit IRDye 800CW secondary antibodies (1:10 000, GE Healthcare, Pittsburgh, PA). Blots were visualized using an ODYSSEY Infrared Imaging System (LI-COR Biosciences, Lincoln, NE) and analyzed using NIH ImageJ software.

### Confocal imaging/analyses of mitochondrial Ca^2+^ uptake during tetanic stimulation

Individual FDB fibers isolated from adult and aged mice were loaded with 5 μM rhod 2-AM for 30 min at room temperature, followed by 4 μM mag-fluo 4-AM for 20 min at room temperature and then exposed to dye-free solution supplemented with 25 μM N-benzylp-toluene sulfonamide (BTS), a skeletal muscle myosin inhibitor, for 20 min to block contractions. Fibers were electrically stimulated with a series of 5 consecutive tetani (500 ms duration, 100 Hz, 0.2 duty cycle) using an extracellular stimulation electrode filled with 200 mM NaCl placed adjacent to the cell of interest. Mag-fluo 4-AM (myoplasmic Ca^2+^) and rhod 2-AM (mitochondrial Ca^2+^) fluorescence signals were monitored. Data were analyzed offline using ImageJ and Igor Pro software. Time series of x-y images (256 × 256 pixels/frame, 0.2 μm/pixel, 690-ms scan duration) were acquired using a Nikon Eclipse C1 Plus confocal microscope (Nikon Instuments) by sequential excitation of green (mag-fluo 4) and red (rhod 2) fluorophores using 488- and 543-nm lasers, respectively. Mag-fluo 4 was monitored during the electrical stimulation, immediately followed by the rhod 2 signal (690 ms after initiation of electrical stimulation). Images were taken at rest, during the 1^st^ and 5^th^ tetanus, followed by imaging every 5 seconds for 2 minutes and then every 30 sec for 8 more minutes. Calculation of mag-fluo 4 fluorescence representing myoplasmic Ca^2+^ levels was performed by calculating the mean intensity of the signal across the fiber during the stimulation. Calculation of the mean rhod-2 fluorescence representing mitochondria located adjacent to CRUs was performed as described previously [17]. Briefly, a 2-μm line was drawn along the longitudinal axis of the fiber loaded (x values corresponding to fiber length and y values corresponding to the fluorescence levels). Rhod-2 values for the difference between profile peaks (I band fluorescence, representing mitochondria) and troughs (A band fluorescence representing background fluorescence) at any given time point following stimulation were normalized to their respective values at rest ([F_Iband_-F_Aband_]_t_/[F_Iband_-F_Aband_]_t=0_). To allow for comparison between different fibers within a given set of experiments, all image acquisition and analysis parameters were kept consistent.

### Mitochondrial membrane potential measurements

FDB fibers from four month old (adult) and 22 month old (aged) C57BL6 mice were isolated as described previously [17] and loaded with 20 nM tetramethylrhodamine methyl ester (TMRE, fluorescent mitochondrial membrane potential indicator) for 20 min at RT. After 20 min, the TMRE concentration was diluted 2-fold and maintained throughout the experiment. To monitor TMRE fluorescence, a time series of images were acquired using a Nikon Eclipse C1 Plus confocal microscope (Nikon Instuments) equipped with a 40X oil objective and 543-nm laser. Sequential images were taken at 15 second intervals. 30 seconds after the start of time series, 1 μM FCCP was perfused onto the fiber while the decay of TMRE fluorescence was continually monitored.

### Statistical analyses

Statistical analyses between means of two groups were conducted using Student's t-test and ANOVA was used to compare means between multiple groups. Statistical significance was set at p<0.01 or at p<0.05, where indicated.

## SUPPLEMENTARY MATERIAL FIGURES AND TABLE



## Abbreviations

ATP: Adenosine triphosphate
Ca^2+^: calcium
CRUs: calcium release units
CM: confocal microscopy
EC coupling: excitation-contraction coupling
EDL: extensor digitorum longus
EM: electron microscopy
FDB: flexor digitorum brevis
ROS: reactive oxygen species
RYR: ryanodine receptor
SR: sarcoplasmic reticulum
TC: terminal cisternae
TT: transverse tubule
WT: wild type
