# Supplementary material for: Age-dependent uncoupling of mitochondria from Ca2+ release units in skeletal muscle
Source: Oncotarget. 2015 Oct 16;6(34):35358–71. doi: 10.18632/oncotarget.6139 (PMC4742110; doi:10.18632/oncotarget.6139)
Supplement: Supplementary file 1 [file oncotarget-06-35358-s001.pdf]

Age-dependent uncoupling of mitochondria from Ca^{2+} release units in skeletal muscle

Supplementary Material

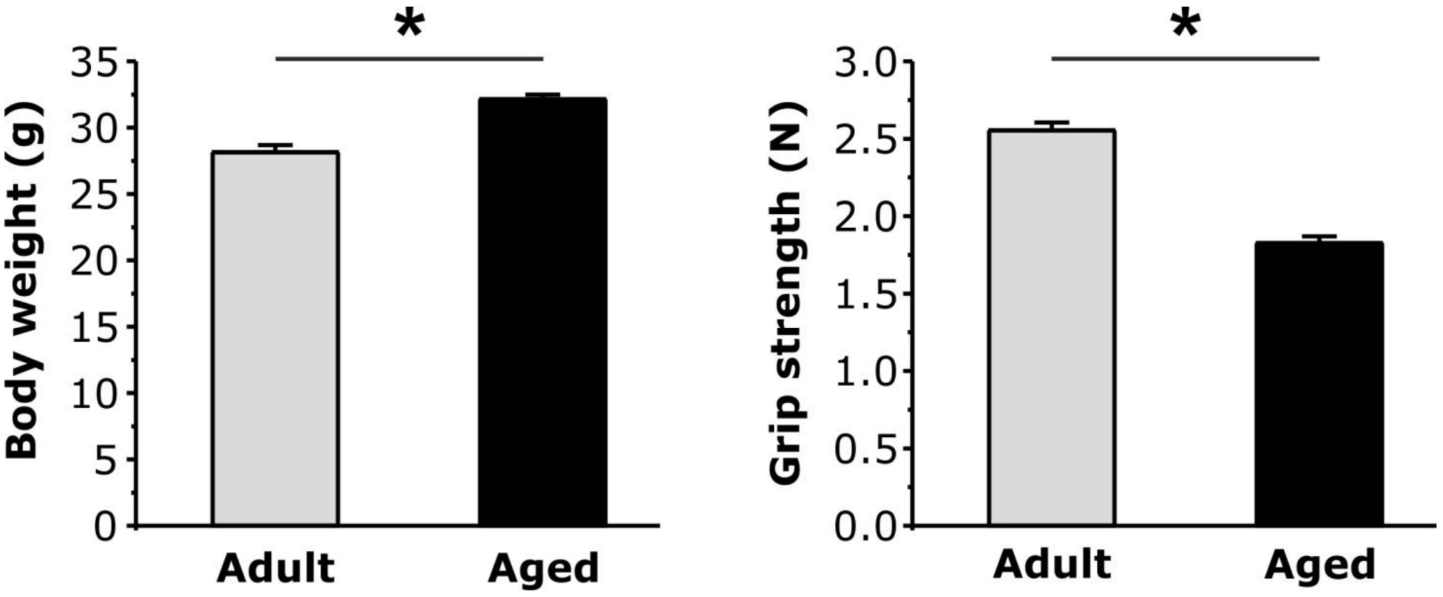

Body weight (g)		Grip strength (N)	
**Adult**	**Aged**	**Adult**	**Aged**
28.2 ± 0.5<br>n=70	32.1 ± 0.3<br>n=64	2.6 ± 0.05<br>n=70	1.8 ± 0.04<br>n=64

Figure S1. Body weight and grip strength in adult and aged mice. Whereas body mass of aged mice is increased compared to that of adult mice (left), maximal grip strength is significantly reduced in aged mice (right). Data are given as means ± SEM (*$p<0.01$); n=number of mice.

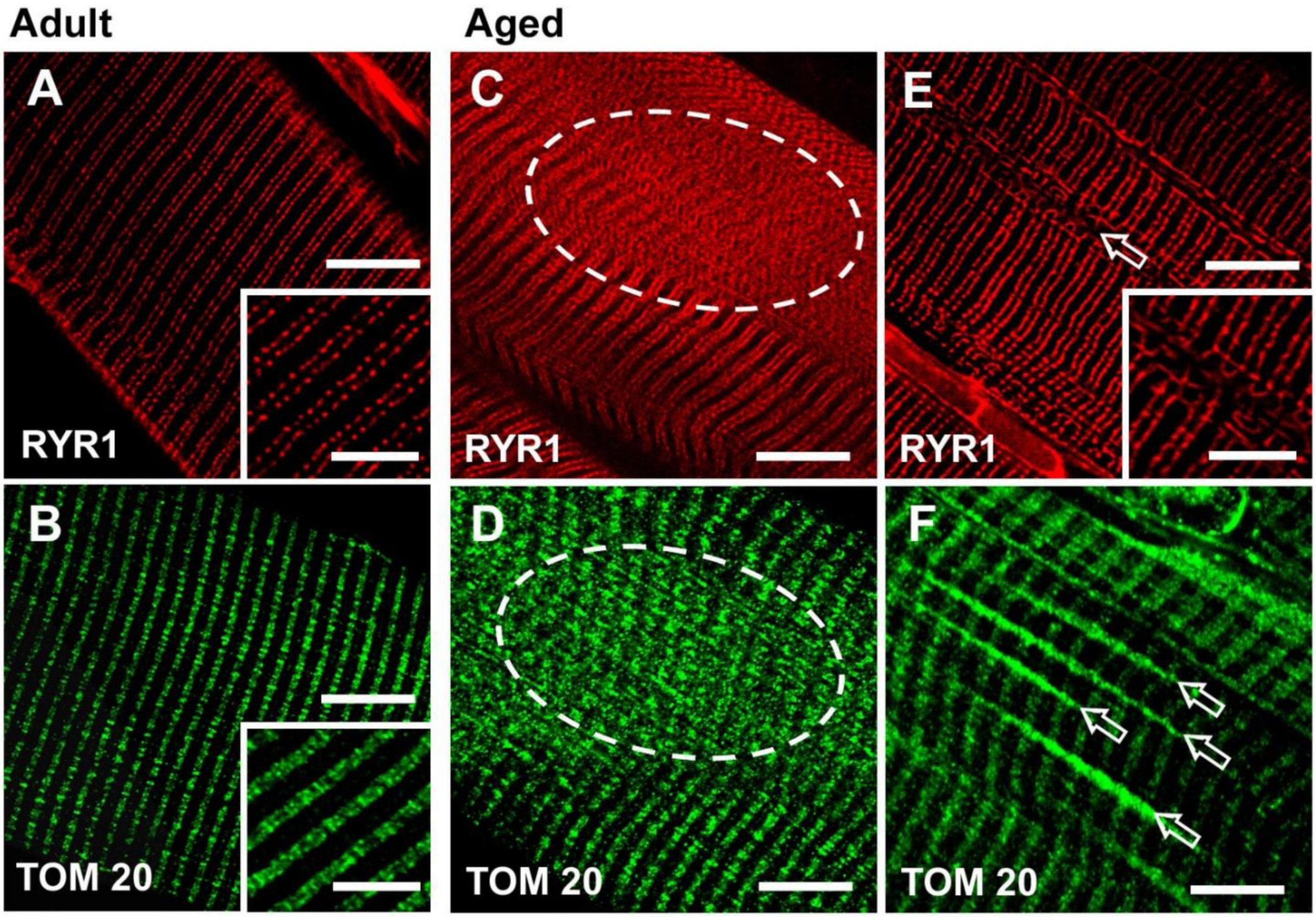

Figure S2. Confocal microscopy of EDL fibers from adult and aged mice immunolabeled with antibodies against RYR1 and TOM-20. A and B) In fibers from adult mice, CRUs and mitochondria are positioned at the A-I band junction of the sarcomere, as shown by precise double-row cross striations (see insets). C-F) In aged mice, this ordered disposition of CRUs and mitochondria is lost in some areas of the fiber (C and D, dashed ovals). In addition, longitudinal rows of mitochondria between myofibrils (F, arrows) are also observed in fibers from aged mice and within these regions, CRUs are misplaced (arrow in E; see also enlarged inset). Scale bars: A-F, 10μm; insets, 5μm.

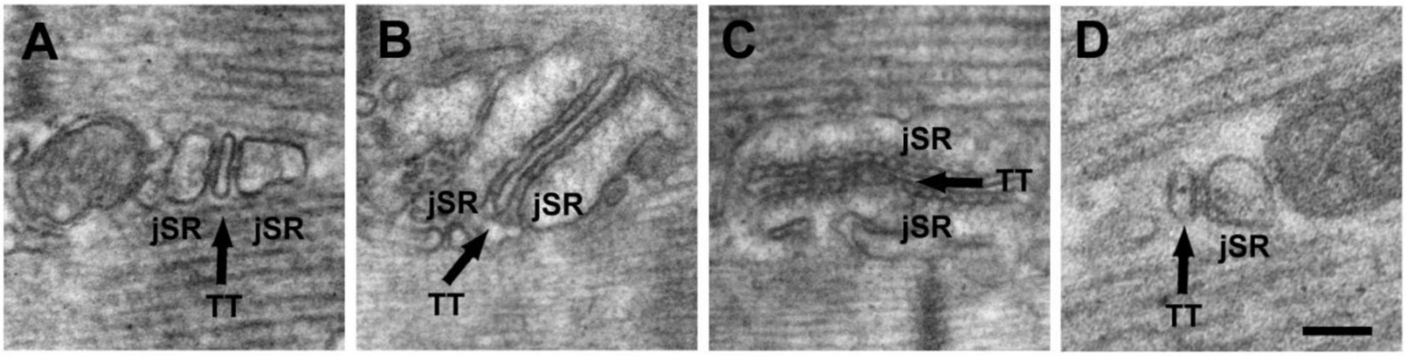

Figure S3. Morphology and orientation of CRUs in fibers from aged mice. A) Normal CRUs are formed by three elements that contain a T-tubule that is oriented transversally with respect to the main axis of the surrounding myofibrils. B-D) In some cases, CRUs are oriented obliquely (B), longitudinally (C), or lack one element (named dyads) (D). See Table 1 for a detailed quantitative analyses. Scale bar: 0.1μm.

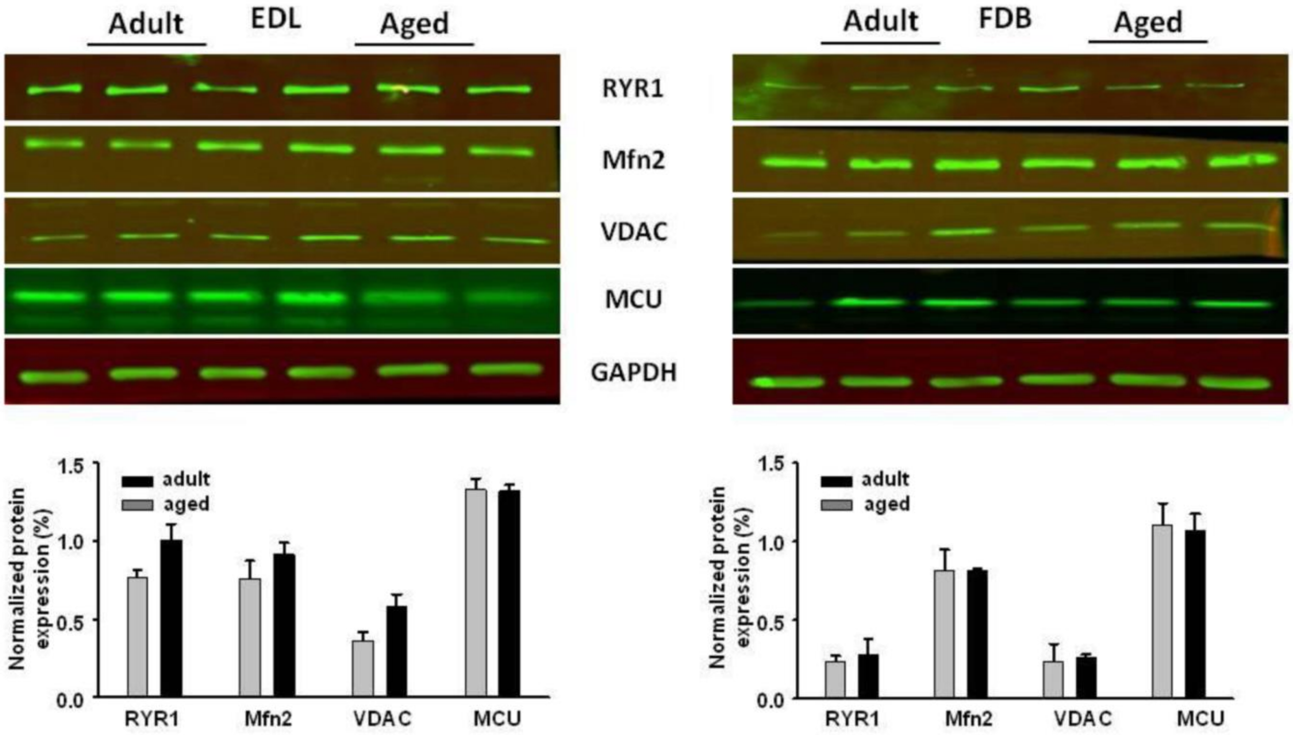

Figure S4. Quantification of expression levels of mitochondrial and Ca^{2+} handling proteins in EDL and FDB muscles from adult and aged mice. A) Representative western blots against RYR1, Mfn2, VDAC, MCU, and GAPDH in EDL muscles from adult and aged mice. B) Representative western blots against RYR1, Mfn2, VDAC, MCU, and GAPDH in FDB muscles from adult and aged mice. Expression levels relative to GAPDH are shown below each series of western blots. Expression of RYR1, Mfn2, VDAC and MCU are not different between EDL muscles from adult and aged mice. Data are plotted as mean ± SEM.

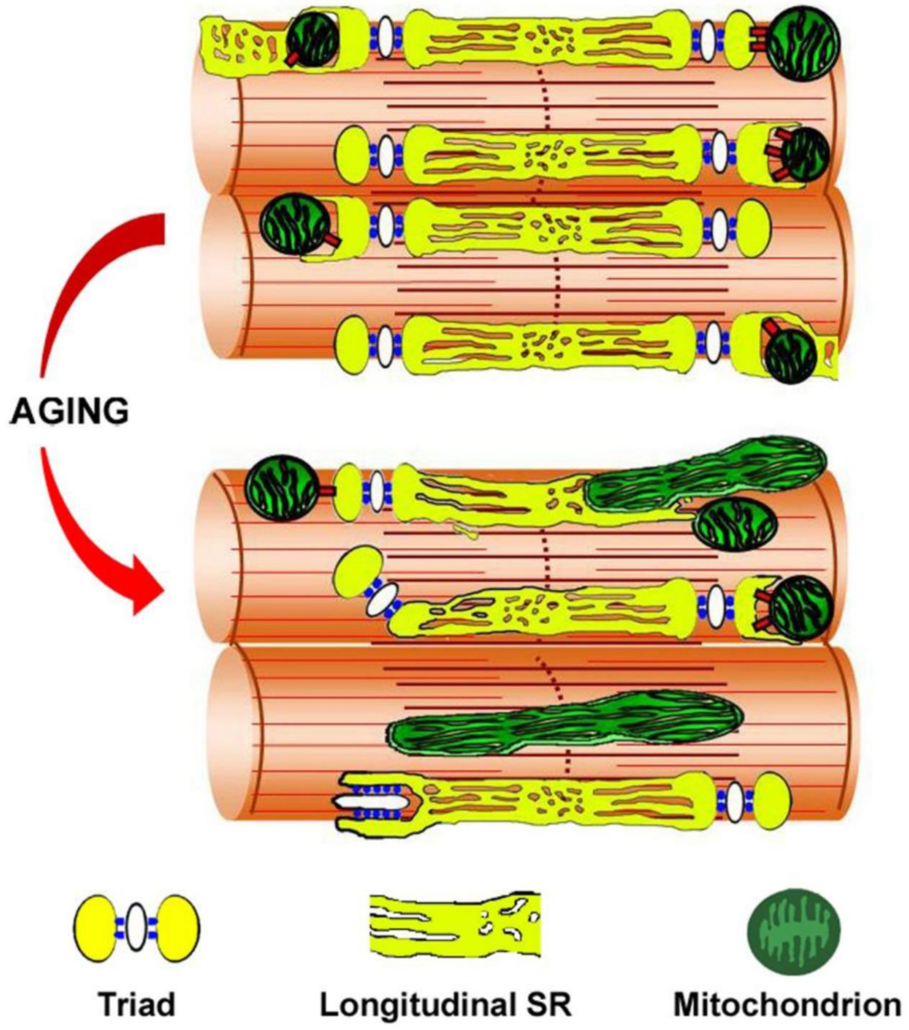

Figure S5. Model summarizing age-related changes to CRUs and mitochondria. The ultrastructural data presented in this manuscript are summarized in this cartoon showing that CRUs and mitochondria exhibit altered orientation and reduced association in muscle from aged mice. Colors legend: SR in yellow; TT in white; mitochondria in green; RYR-feet in blue; tethers in red; myofibrils in orange.

Table S1. Quantitative analyses of density of tethers and distance between CRUs and mitochondria.

	A	B	C	D
	**No. of tethers /100 Mito-CRU pairs**	**No. of tethers /100 μm^2**	**Minimum average distance between SR and outer membrane (nm)**	**No. of Mito-CRU pairs /100 μm^2**
**Adult**	35.3 ± 0.2	13.8 ± 0.4	15.7 ± 0.9	37.4 ± 0.8
**Aged**	23.0 ± 0.1*	5.5 ± 0.3*	22.2 ± 1.4*	27.0 ± 0.7*

*Data are shown as mean ± SEM (*p < 0.01).*
Samples size: 40 fibers from 5 Adult mice and 40 fibers from 5 Aged mice; 10 micrographs/fiber.

The number of tethers observed in 100 mitochondria-CRU pairs (column A) and the number of tethers / 100μm^2 (column B) is significantly decreased in fibers from aged mice. The average minimum mitochondrion-SR distance is increased in fibers from aged mice (column C), whereas the number of CRU-mitochondrion pairs / 100μm^2 is significantly decreased (column D). See Fig. 2 for additional detail.
